# Host shifts and molecular evolution of H7 avian influenza virus hemagglutinin

**DOI:** 10.1186/1743-422X-8-328

**Published:** 2011-06-28

**Authors:** Camille Lebarbenchon, David E Stallknecht

**Affiliations:** 1 Southeastern Cooperative Wildlife Disease Study, Department of Population Health, College of Veterinary Medicine, The University of Georgia, Athens, Georgia 30602, USA

**Keywords:** Influenza A, duck, poultry, adaptation, virulence, receptor binding domain, parallel evolution

## Abstract

Evolutionary consequences of host shifts represent a challenge to identify the mechanisms involved in the emergence of influenza A (IA) viruses. In this study we focused on the evolutionary history of H7 IA virus in wild and domestic birds, with a particular emphasis on host shifts consequences on the molecular evolution of the hemagglutinin (HA) gene. Based on a dataset of 414 HA nucleotide sequences, we performed an extensive phylogeographic analysis in order to identify the overall genetic structure of H7 IA viruses. We then identified host shift events and investigated viral population dynamics in wild and domestic birds, independently. Finally, we estimated changes in nucleotide substitution rates and tested for positive selection in the HA gene. A strong association between the geographic origin and the genetic structure was observed, with four main clades including viruses isolated in North America, South America, Australia and Eurasia-Africa. We identified ten potential events of virus introduction from wild to domestic birds, but little evidence for spillover of viruses from poultry to wild waterbirds. Several sites involved in host specificity (addition of a glycosylation site in the receptor binding domain) and virulence (insertion of amino acids in the cleavage site) were found to be positively selected in HA nucleotide sequences, in genetically unrelated lineages, suggesting parallel evolution for the HA gene of IA viruses in domestic birds. These results highlight that evolutionary consequences of bird host shifts would need to be further studied to understand the ecological and molecular mechanisms involved in the emergence of domestic bird-adapted viruses.

## Background

Over the last decade, an increasing number of studies has focused on the effects of human activities on pathogen evolution [1,2]. Historically, agriculture and domestication of wild animals have been linked to the emergence of several human pathogens. Ecological changes related to modern agricultural practices also are likely to affect emergence of both human and animal diseases [3,4]. Influenza A (IA) virus provides a good example of a pathogen that can move from wild bird reservoir to domestic animal systems and adapt to humans and other mammals. These viruses have invaded, and in some cases have become established in a diversity of agrosystems, ranging from integrated rice-duck farming [5], to live poultry markets [6] and industrial and intensive farming systems [7,8]. The emergence and long-term circulation of the highly pathogenic (HP) H5N1 virus in domestic birds in Southeastern Asia [9,10], as well as the recent emergence of the swine-origin H1N1 virus in humans [11,12], highlights the ability of IA viruses to spread beyond species barriers and adapt rapidly to new host and environmental conditions [13].

Wild waterbirds in the orders Anseriformes (ducks, geese and swans) and Charadriiformes (gulls, terns and waders) are recognized to be natural hosts for low pathogenic (LP) IA viruses [14]. In these two avian orders, a large diversity of IA subtypes have been described, based on genetic and antigenic characteristics of the hemagglutinin (HA) and the neuraminidase (NA) proteins. In wild waterbirds, IA viruses do not cause significant disease but may have subtle physiological and behavioral effects [15,16]. In ducks, ecological factors affecting the prevalence of infection include host species, age, behavior, population density, and persistence of viruses in the environment.

In domestic birds (e.g. chickens, ducks, turkeys, quails), IA viruses replicate in both the intestinal and respiratory tracts, but in the case of HP H5 and H7 viruses, can result in multi-organ systemic infections with high mortality [17,18]. Several HP IA virus outbreaks have been described and were responsible for large economic losses for poultry producers [18]. With the exception of the Asian strains of the HP H5N1, HP viruses are rarely reported in wild waterbirds [19,20].

Genetic exchanges between viruses circulating in wild and domestic birds have been documented, especially when wild bird origin LP viruses infect poultry [21-23]. The regular spillover of Asian HP H5N1 viruses from domestic to wild birds also demonstrate the potential for reverse flow [24-26]. However, the time, location, frequency, effect on the population genetic diversity, and on the molecular evolution of IA viruses of such genetic exchanges remain unclear. The evolutionary consequences of these host and resulting environmental shifts represent a challenge to understand the mechanisms involved in the emergence of IA viruses, in particular regarding molecular changes involved in the increase of virulence and host specificity.

We focused on the evolutionary history of H7 IA viruses circulating in wild and domestic birds. Compared to other HA subtypes, numerous H7 viruses have been isolated from both wild waterbirds and poultry. In domestic birds, H7 IA viruses have been responsible for severe outbreaks sometimes with long-term circulation of LP and HP viruses; in Italy [21,27-29], Germany [30], the Netherlands [31], Australia [32,33], China [34], Pakistan [35], Canada [36,37], United States of America (USA) [38,39] and Chile [40].

The aims of this study were: (i) to provide an overview of subtype combinations, host diversity and phylogeographic structure of H7 IA virus isolated in wild and domestic birds, worldwide; (ii) to investigate the population dynamic of H7 HA, with a particular emphasis on virus emergence and extinction dates, genetic diversity and exchanges between wild and domestic hosts; and (iii) to estimate changes in nucleotide substitution rates and test for positive selection in the HA gene. We discuss the consequences of host shifts on the population dynamics of H7 IA viruses in wild and domestic birds and the ecological and molecular mechanisms potentially involved in the emergence of domestic bird-adapted viruses.

## Methods

### Sequence dataset

Complete nucleotide and protein sequences of the HA gene were downloaded from the Influenza Sequence Database (ISD; [41]), on October 22, 2009. For each sequence, the following information was collected: accession number, strain name, subtype, geographic origin (i.e., continent: Europe, Asia, Australia, Africa, North America and South America), bird host species, virulence (LP or HP) and year of virus collection. Host status (domestic or wild) was determined based on bird species or from the description of the isolate provided in reference papers. Sequences from viruses for which host was not identified (e.g., 'duck' or 'mallard', without any additional information concerning their origin) were not included in the analyses. Duplicate sequences (from the same strain) as well as sequences previously identified as reflecting potential laboratory errors [42] also were removed. When not available in the ISD, virulence (LP or HP) of viruses was assigned by comparing the HA amino acid sequence pattern of the cleavage site with viruses previously characterized as LP or HP. Differences in the frequency of subtype combinations between wild and domestic birds were investigated using a χ^2^ test implemented in the R 2.10.1 software [43].

### Phylogeographic analysis

The coding region of nucleotide sequences was aligned with ClustalW 2.0.10 [44]. Four viruses representing two different subtypes (H3N8 and H6N2) and geographic origins (North America and Europe) were included to root trees: A/Green-winged teal/Ohio/1289/2005 (H3N8), A/Mallard/Maryland/2022/2005 (H6N8), A/Mallard/Finland/12072/06 (H3N8) and A/Bewick's swan/Netherlands/1/2005 (H6N2). Phylogenetic trees were constructed by maximum likelihood with the software PhyML 3.0 [45]. The evolutionary model was selected by Model Generator 0.85 [46]. Nodal supports were assessed with 100 bootstrap replicates.

### Population dynamics

Viral population dynamics were investigated with a Bayesian Markov Chain Monte Carlo coalescent approach, implemented in the program BEAST 1.5.3 [47]. Time of the most recent common ancestor (TMRCA) as well as rates of nucleotide substitution were obtained from analyses performed with BEAST. The Shapiro-Rambaut-Drummond-2006 (SRD06) nucleotide substitution model was used in all simulations as this model is recognized to provide better resolution for coding regions [48], and has recently been used in population dynamic studies of other IA subtypes [49,50].

We focused on the viral population dynamics of two main genetic lineages identified by the phylogeographic analysis (cf. results section): (i) the North American-South American lineage (NA-SA) and (ii) the Eurasian-African-Australian lineage (EURAS-AF-AU). Three molecular clock models were tested for each genetic lineage. The strict clock (SC) that assumes a single evolutionary rate in the phylogenetic trees, and two relaxed clocks: the uncorrelated exponential (UE) and the uncorrelated lognormal (UL), which allow evolutionary rates to vary along branches, within an exponential or lognormal distribution [51]. Molecular clock models were evaluated and tested with the Bayes Factor (BF) [52,53] implemented in the program TRACER 1.5.0 [47,54]. The ratio of marginal likelihoods were compared between models and BF significance was determined from the values of 2ln(BF), as described by Brandley *et al*. [55]. A Bayesian skyline coalescent tree prior was used in all simulations as it makes the fewest a priori assumptions about the data [56] and has been shown to be more appropriate to describe the population dynamics of IA virus [57]. Analyses were performed (i.e. with SC, UE and UL molecular clocks) for each genetic lineage, with a chain length of 120 million generations sampled every 1 000 iterations. Results were analyzed with TRACER: for each simulation, the first 5-10% trees were discarded as burn-in and an effective sample size superior to 200 was obtained to ensure adequate sample size for the posterior, prior, likelihood, mean rate of nucleotide substitution (clock rate for SC), and skyline. Phylogenetic consensus trees were produced using the program TREEANNOTATOR 1.5.3 and edited for generation of figure captions with the program FigTree 1.3.1.

We also performed independent analyses including only viruses isolated in wild or domestic birds for each genetic lineages (NA-SA and EURAS-AF-AU) and for the main genetic sub-lineages (we limited the analysis to groups including more than ten sequences obtained from viruses isolated over a time period of at least a three years). Analyses were carried out with a Bayesian skyline coalescent tree prior and a UE molecular clock (most appropriate according to the results of the previous analysis).

### Molecular evolution of the HA gene

Signatures of selection in the HA nucleotide sequences were evaluated using the codon-based approach as implemented in HyPhy [58], available at the Datamonkey webserver [59,60]. Estimations of the d_N _(nonsynonymous substitutions) and d_S _(synonymous substitutions) were obtained with the single likelihood ancestor counting (SLAC), fixed-effects likelihood (FEL) and random effects likelihood (REL) methods [61]. Analyses were conducted under the HKY85 nucleotide substitution model, with a statistical significance set to p = 0.05. Finally, in order to identify the potential function of positively selected amino acids, we located N-linked glycosylation sites in the HA protein sequences using the NetNGlyc 1.0 Server.

## Results

### Dataset

Among the 508 sequences downloaded from the ISD, 94 (18.5%) were excluded from the analysis because: (i) they represented duplicate sequences of the same virus (N = 21), (ii) they were previously identified as potential laboratory errors (N = 3; [42]) or (iii) information concerning the bird species and/or its status (domestic or wild) was not available (N = 70).

The overall proportions of viruses isolated from domestic and wild birds were 84% and 16%, respectively. The most represented bird species was chicken (53%) for domestic birds and Mallard (41%) for wild birds (Additional file 1: Figure S1). Ninety percent of isolated viruses came from North American and European birds (Additional file 2: Table S1). Subtype combinations with the NA were significantly different between domestic and wild birds (χ^2^ = 71.6, df = 6, p <0.001, excluding N6 and N8; cf. Additional file 3: Figure S2 for details). For wild birds, H7N3 and H7N7 were the most represented subtypes (49% and 35% respectively), while in domestic birds H7N2 was mainly represented (52%), with H7N3 (20%) and H7N1 (19%). Seventy-six viruses were HP (18%); all of them were isolated from domestic birds (Additional file 2: Table S1).

### Phylogeographic structure

**Figure 1:**
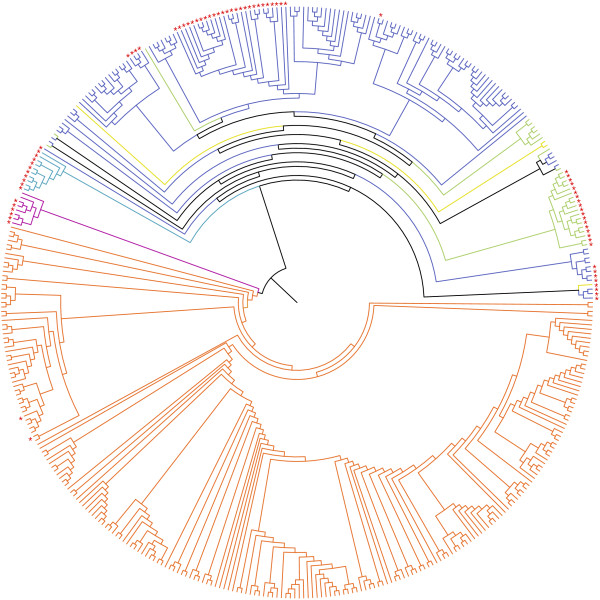
**Maximum likelihood consensus tree derived from 414 H7 HA nucleotide sequences**. Computations were realized with the GTR+I+Г evolutionary model and 100 bootstrap replications (I = 0.27; α = 0.85). Tip colors represent the geographic origin of viruses: dark blue: Europe; light blue: Australia; green: Asia; yellow: Africa; purple: South America; orange: North America. Internal branches were also colored for monophyletic groups (i.e., same continent). Tips were annotated with red stars for HP viruses. A detailed phylogenetic tree including virus strain names is available in Figure S3.

A strong association between the geographic origin and genetic structure was observed, with four main clades including viruses isolated in (i) North America, (ii) South America, (iii) Australia and (iv) Europe, Asia and Africa (Figure 1 and Additional file 4: Figure S3). The two latter groups were less supported by bootstrap values; however, they were excluded from a 'superclade' including all American viruses (North and South America; Additional file 4: Figure S3). Several sub-groups were observed in each geographic clades, including viruses isolated in the same location or time period (Additional file 4: Figure S3 for details). Most of these clades have previously been identified in studies that focused on a limited number of viruses, responsible for outbreaks in poultry, locally. Finally, we observed that HP H7 viruses isolated in domestic birds in Eurasia-Africa, Australia, North and South America formed unrelated genetic lineages.

### Evolutionary history

**Figure 2:**
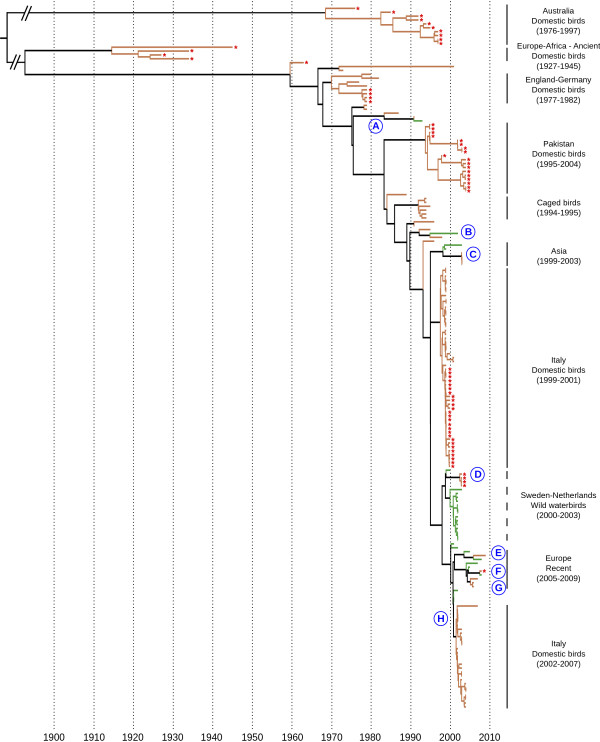
**Maximum clade credibility tree for the Eurasian-African-Australian genetic lineage**. Tip and branch colors represent host origin (wild in green, domestic in orange). Tips were annotated with red stars for HP viruses. Blue letters (A to H) represent host shift events. Main genetic lineages, with information related to virus origin, are highlighted in the right part of the trees. Except for the ones identified with dashed lines, these lineages were monophyletic and supported by posterior probability values equal to 1. A detailed tree, including strain names, posterior probability values and 95% highest posterior density for time of the most recent common ancestors, is available in Figure S4.

**Figure 3:**
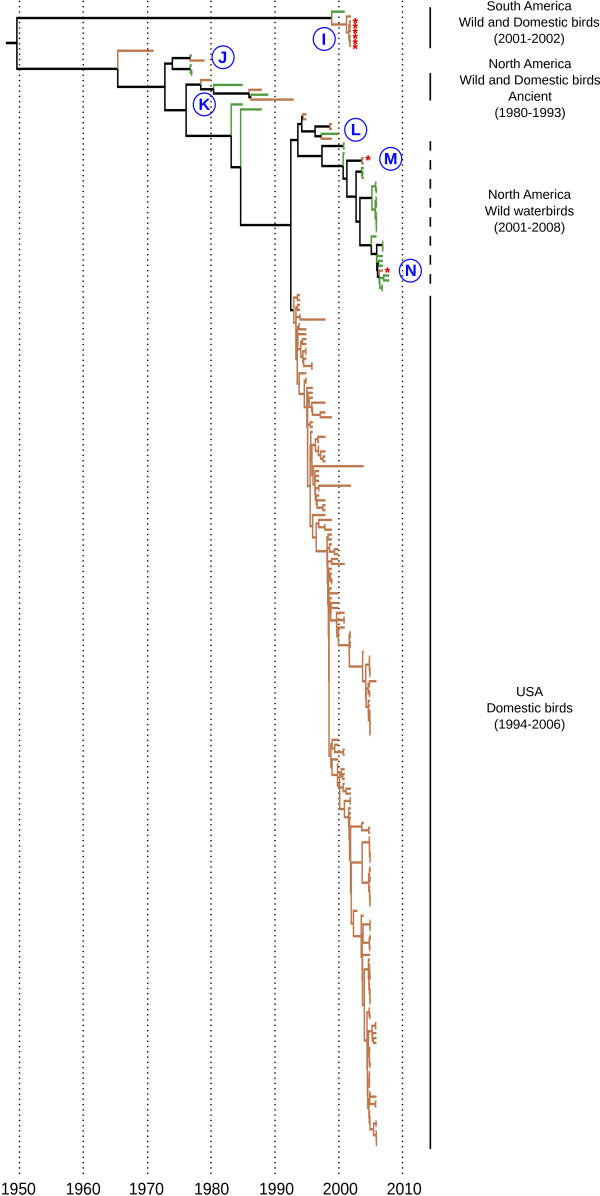
**Maximum clade credibility tree for the North-South American genetic lineage**. Tip and branch colors represent host origin (wild in green, domestic in orange). Tips were annotated with red stars for HP viruses. Blue letters (I to N) represent host shift events. Main genetic lineages, with information related to virus origin, are highlighted in the right part of the trees. Except for the ones identified with dashed lines, these lineages were monophyletic and supported by posterior probability values equal to 1. A detailed tree, including strain names, posterior probability values and 95% highest posterior density for time of the most recent common ancestors, is available in Figure S5.

The North American-South American (NA-SA) and the Eurasian-African-Australian (EURAS-AF-AU) lineages were analyzed independently, because of their strong genetic divergence as well as for computational reasons. For both lineages, the UE molecular clock model was significantly better than the SC and UL models, concordant with other studies [49,50]. Based on the maximum clade credibility tree topologies, we identified at least 14 potential events of host shift (Figures 2 &3 and Additional file 5 &6: Figures S4 & S5 - events A to N). Ten of these corresponded to viruses that circulated in domestic birds and shared a common ancestor with a virus isolated from wild birds prior to the detection in domestic birds (i.e. introduction from wild to domestic birds; Additional file 7: Table S2). The direction of virus introduction (i.e. wild-to-domestic or domestic-to-wild) for the four other host shift events (A, B, K and L; Figures 2 &3 and Additional file 5 &6: Figures S4 & S5) couldn't be determined because of the limited number of sequences available (3 to 6), sometimes over extended time periods (2 to 13 years).

The EURAS-AF-AU lineage was structured into ten major genetic sub-lineages, strongly supported by posterior probability values (Figure 2 and Additional file 5: Figure S4). The Australian H7 viruses were clearly differentiated from Eurasian-African viruses and the time of the most recent common ancestor (TMRCA) between the two lineages was 1873 (95% HPD: [1811-1914]). The TMRCA estimated for the Australian lineage was 1968 [1952-1975] with a circulation of HP viruses for more than 29 years in domestic birds, and genetic reassortment with different NA subtypes.

The Eurasian-African (EURAS-AF) genetic lineage was divided into 9 major clades including viruses isolated in different geographical locations and time period (Figure 2 and Additional file 5: Figure S4). The presence of a sub-lineage including viruses isolated between 1927 and 1945 suggested an ancient circulation of genetically differentiated HP H7 IA virus in domestic birds in Europe and Africa. These viruses did not shown genetic relatedness with modern HP H7 IA viruses and could have circulated in domestic birds for 21 to 48 years in Europe and Africa (TMRCA: 1912 [1897-1924]). The existence of such an ancient lineage of H7 HA viruses has been suggested by other studies; however, our results demonstrate that they are included in the EURAS-AF genetic lineage and are not at the basis of the EURAS-AF-AU lineage, as previously suggested (e.g. [62]).

A lineage including LP and HP viruses isolated in England and Germany, between 1977 and 1982, could have emerged in domestic birds in 1972-1973 (TMRCA: 1973 [1969-1976]). This lineage is probably extinct as no genetically related H7 HA viruses have been detected after 1982. In addition, the genetic structure of this clade supports the circulation of two sub-lineages of LP H7 in England, one at the origin of the HP H7N7 outbreak in domestic birds in Germany in 1979 (A/Turkey/England/647/1977) as previously reported by Banks *et al*. [62].

A distinct clade of HP H7N3 viruses was evidenced with domestic birds in Pakistan, with a co-circulation of two genetically different sub-lineages. According to the TMRCA of this clade (1993 [1991-1995]), HP H7 viruses could have circulated for 11 years in domestic birds. These viruses were genetically unrelated to other HP H7 viruses circulating during the same time period in Europe (Italy, Germany and the Netherlands), suggesting an independent emergence and circulation, as recently proposed by Abbas *et al*. [35].

Two unrelated lineages were observed in Italy, in domestic birds. The first one included both LP and HP H7N1 viruses that circulated between 1999 and 2001, with a strong clustering of HP viruses (TMRCA: 1997 [1996-1998]). The second one included only LP H7N3 viruses isolated between 2002 and 2007 (TMRCA: 2001 [2001-2002]). Our analysis also supported the introduction of LP H7N3 from wild birds to domestic poultry in Italy (host shift H; cf. [21] for complete description).

A paraphyletic group including H7 IA viruses isolated from wild birds, in Sweden and the Netherlands was also observed (TMRCA: 1999 [1999-2000]). A genetic clade including HP viruses isolated from domestic birds was branched within this group (TMRCA: 2002 [2002-2003]). As reported by Munster *et al*. [22], the HA of A/Mallard/Netherlands/12/2000 was genetically related to the four HP H7 HA from domestic birds in the Netherlands and Germany in 2003 (Additional file 5: Figure S4), suggesting that this HP H7 outbreak could have arisen from the circulation of LP H7 IA viruses in wild waterbirds (host shift D).

We revealed the existence of a genetic lineage recently isolated from both wild and domestic birds in Europe. Two sub-lineages were identified and provided evidence of virus transmissions from wild to domestic birds (host shifts E, F and G). For both sub-lineages (including: A/Mallard/Italy/299/2005 and A/Mute swan/Hungary/5973/2007; cf. Additional file 5: Figure S4 for details) the TMRCA was 2004 [2003-2005].

In Asia (China and Japan), we identified a clade that includes viruses isolated from wild and domestic birds. This result suggested a regional circulation of genetically differentiated H7 viruses, with a likely host shift from wild to domestic birds (host shift B). The TMRCA of this clade (1998 [1997-1999]), together with the detection of viruses in domestic birds in 2003, suggest that this genetic sub-lineage could have circulated in domestic birds in China for five years.

Finally, we highlighted the existence of two 'atypical' genetic clades. The first included H7N1 IA viruses isolated in caged birds in Asia and Europe, between 1994 and 1995 (Figure 2 and Additional file 5: Figure S4). The geographical origin of these genetically related viruses highlights the potential for rapid spread of H7 IA viruses due to illegal caged bird trade (cf. [62] for discussion). The second clade included two viruses: A/Parrot/Northern Ireland/VF7367/1973 and A/Chicken/Chakwal/NARC-35/2001 (Additional file 5: Figure S4). The isolation of these viruses from distant geographic areas (Northern Ireland and Pakistan) and over a 28 years period, could support a long term circulation of an undetected H7 lineage in domestic birds, in Eurasia (cf. [35] for discussion). However, given the extremely high level of identities between the two HA sequences (99% homology) they probably reflect laboratory errors (cf. [42] for discussion).

**Figure 4:**
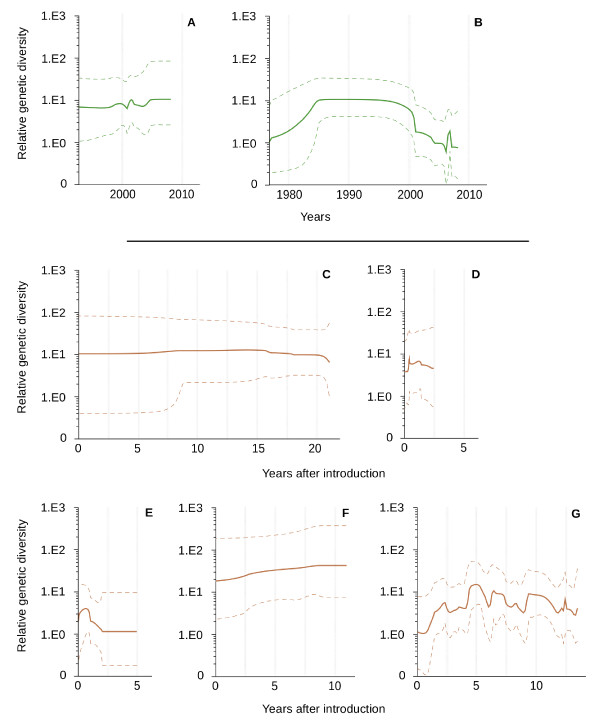
**Relative genetic diversity over time for the H7 HA of IA viruses circulating in wild (A, B) and domestic (C-G) birds**. A: wild birds Eurasia-Africa-Australia; B: North and South America; C: Australia; D: Italy (1999-2001); E: Italy (2002-2007); F: Pakistan; G: USA. Dashed lines represent 95% highest posterior density.

Temporal changes in genetic diversity were plotted independently for viruses isolated in wild and domestic birds, with a Bayesian Skyline reconstruction (Figure 4). Although not significant, the recent co-circulation of several genetically different lineages in wild birds was illustrated by a slight increase in the relative genetic diversity in 2001 and after 2004 (Figure 4A). After introduction in domestic birds, no significant changes in the relative genetic diversity were observed (Figure 4C, 4D, 4E, 4F) although these patterns may reflect the low number of sequences available for certain genetic lineages (e.g. Australia).

The NA-SA genetic lineage was also structured into sub-lineages (Figure 3 and Additional file 6: Figure S5). Viruses isolated from North and South America were strongly differentiated (TMRCA: 1954 [1928-1969]). The TMRCA of the South American sub-lineage was 1999 [1997-2001] and included a LP H7N3 virus isolated in a wild bird (A/Cinnamon Teal/Bolivia/4537/2001), presumably at the origin of the circulation of LP and HP H7N3 viruses detected in domestic birds in Chile during the following year (host shift I; cf. [40] for discussion).

The North American lineage was divided into three major sub-lineages. An ancient sub-lineage included viruses isolated from wild and domestic birds, between 1980 and 1993. Based on the TMRCA (1979 [1977-1980]), this genetic sub-lineage circulated for at least 14 years in the USA and Canada. Several host shifts would have occurred (K), however the direction of virus introductions (i.e. wild-to-domestic or domestic-to-wild) remains to be determined.

A second genetic sub-lineage included LP H7 IA viruses isolated from wild and domestic birds in North America, between 2001 and 2008. Two genetic groups were observed and suggested the co-circulation of genetically different H7 IA viruses in wild waterbirds between 2005 and 2006 (TMRCA: 2005 [2005-2006] for both clades, including A/Ruddy Turnstone/Delaware/752/2006 and A/Blue-winged Teal/Ohio/566/2006, respectively; Figure S5). Genetically related viruses isolated in domestic birds suggested two host shifts (M and N; Figure 3, Additional file 6: Figure S5 and Additional file 7: Table S2), with a rapid evolution toward an increased virulence (cf. [37,63] for discussion).

We underlined the long-term circulation of H7 HA in domestic birds in the USA and dated the emergence of this sub-lineage in 1992-1993 (TMRCA: 1993 [1989-1994]). These viruses would have circulated for 13 years in domestic birds with co-circulation of genetically different viruses at the same time and location (e.g. in Chicken in New-York in 2005; Additional file 6: Figure S5).

In wild birds, temporal changes in the relative genetic diversity showed an important decrease after 2000, and a recent peak in 2006 (Figure 4B). In the USA, after introduction in domestic birds, important fluctuations were observed from 1992 onward with peaks of diversity reached every 2-3 years (Figure 4G), although these changes were not significant.

### Molecular evolution

Mean nucleotide substitution rates were calculated for viruses isolated in wild and domestic birds, separately, for each major genetic lineage (NA-SA and EURAS-AF-AU) and sub-lineages (Table 1). Overall, high rates of substitutions were obtained, ranging from 0.37 × 10^-3 ^to 11.74 × 10^-3 ^substitution/site/years. Higher rates were obtained for viruses circulating in wild birds than for those isolated from domestic birds. Differences were also obtained in domestic birds between genetic lineages.

**Table 1:** Molecular evolution of the HA gene for the main genetic lineages and sub-lineages of H7 IA viruses

Geographic origin	Host	N^1^	Time period^2^	Mean nucleotide substitution rate^3^	Virulence	Mean d_N_/d_S _[95% CI]	Positive selection^4^			Amino acid position
							SLAC	FEL	REL	
Eurasia-Africa-Australia	W	30	25	4.32 [2.24-6.47]	LP	0.09 [0.08-0.11]	0	0	0	
*Sweden - Netherlands*	W	15	4	11.21 [0.23-21.14]	LP	0.18 [0.13-0.25]	0	0	0	
Eurasia-Africa-Australia	D	151	144	2.25 [1.59-2.86]	LP-HP	0.13 [0.12-0.14]	2	1^5^	NA	143, 341
*Italy*	D	52	4	2.99 [1.26-4.90]	LP-HP	0.44 [0.34-0.55]	0	1^5^	1	143
*Italy*	D	27	6	5.59 [3.99-7.33]	LP	0.27 [0.21-0.35]	0	0	0	
*Pakistan*	D	18	11	0.37 [0.06-0.69]	HP	0.73 [0.53-0.99]	0	1^5^	4	46, 139, 143, 152
*Australia*	D	10	29	4.62 [2.78-6.28]	HP	0.11 [0.09-0.15]	0	2^5^	2	150, 284

North America	W	37	32	6.59 [4.93-8.28]	LP	0.09 [0.07-0.11]	0	0	0	
*North America*	W	28	9	11.74 [6.55-17.88]	LP	0.10 [0.07-0.12]	0	0	0	
North and South America	D	195	59	3.87 [2.82-4.79]	LP-HP	0.16 [0.15-0.17]	1	1 + 1^5^	NA	150, 340
*USA*	D	175	13	4.32 [3.66-5.01]	LP	0.24 [0.21-0.26]	1^5^	3	NA	143, 148, 276

^1 ^number of sequences; ^2 ^number of years of circulation between the time of the most recent common ancestor of the genetic lineage and the last time of virus collection; ^3 ^per 10^-3 ^substitution/site/year, with [95% HPD]; ^4 ^number of sites under positive selection, with p <0.05, according to the single likelihood ancestor counting (SLAC), fixed-effects likelihood (FEL) and random effects likelihood (REL) methods; ^5 ^evidence of positive selection for the same site(s), as found with another method, but with 0.1 <p <0.05. D: domestic bird; W: wild bird; LP: low pathogenic; HP: highly pathogenic; CI: confidence interval; NA: not available because of computational limitations.

We tested evidence of positive or negative selection for the same genetic lineages (Table 1). For viruses isolated in wild birds, a strong evidence of purifying selection was found with low mean d_N_/d_S _ratio and numerous negatively selected sites. When all viruses isolated from domestic birds were considered, for each geographic lineage (EURAS-AF-AU and NA-SA), strong evidence of purifying selection also was found (i.e. sites under negative selection); however several sites were detected to be under positive selection. When analyzing the main genetic sub-lineages independently (Table 1), high d_N_/d_S _ratios were also found, with a lower number of sites under negative selection and evidence for positive selection for several amino acids.

The majority of positively selected sites were located in the HA1 coding region of the HA (position 1 to 339, H7 HA numbering; Table 1). Only two amino acids, at positions 340 and 341, were located outside the HA1 coding region, in the cleavage site of the protein. We determined the N-linked glycosylation sites in the HA protein for viruses isolated in wild birds. Three sites were identified (position 30, 46 and 249) in the HA1 coding region, for 100% of the sequences analyzed. Among the positively selected sites in domestic birds, we found that the amino acid mutation at position 143 (from Ala, Val or Lys, to Thr) led to the generation of a fourth sequon (^140-^Asn-Gly-Thr^-144^) in the HA1 coding region. In wild birds, only one of the 68 sequences analyzed showed the presence of this fourth sequon (A/Northern shoveler/California/HKWF1026/2007). For domestic bird viruses, there were also evidence of positive selection for amino acid changes leading to the removal of a highly conserved sequon, at position 46 (^45-^Asn-Ala-Thr^-49 ^to ^45-^Asp-Ala-Thr^-49^). This observation needs to be confirmed as only one of the three methods detected evidence of positive selection and the analysis was based on a low number of sequences (Table 1). The consequence of amino acid mutation at other sites under positive selection is not clear, although most were located in, or at the edge of, the receptor binding domain of the HA protein (positions 139, 148, 150 and 152).

## Discussion

In this study, we provided an extensive phylogenetic analysis of 414 H7 IA viruses that have been isolated worldwide in wild and domestic birds, and investigated the consequences of host shifts on the molecular evolution of the HA gene. First, we underlined that precise information related to host is critical. The lack of accuracy related to the species or 'host status' (domestic or wild) in public sequence databases limits our ability to effectively study host shifts and their consequences on viral evolution. We indeed excluded 14% of the sequences composing the initial dataset because of missing or unclear information related to the bird species or status. In the context of increasing isolations of IA virus and availability of sequences, this issue needs immediate attention.

### Evolutionary history of H7 IA virus in wild birds

The overall phylogenetic structure of H7 HA was concordant with results reported in previous studies, based on a limited number of sequences (e.g. [62]): a clear phylogeographic pattern was evidenced with distinct genetic lineages in the Eastern and Western hemispheres. High divergence was observed, in particular for the Australian and South American clades, but also at a smaller spatial scale for viruses responsible for outbreaks in domestic birds. Genetic exchanges between biogeographical regions have been documented for IA viruses, especially between North America and Europe, and North America and Asia (e.g. [64]). These exchanges are likely to be favored by habitat sharing in breeding areas between waterbirds using different migratory flyways, and can have important consequences on the population dynamics of IA viruses in wild birds, such as the replacement of endemic genetic lineages [50]. For H7 IA viruses, the strong phylogeographic structure supports that no intercontinental exchanges resulting in the replacement of an endemic lineage have occurred recently between Eurasia and the Americas.

Regular emergence and co-circulation of genetically different H7 IA virus lineages were identified in wild waterbird populations. In Eurasia, the circulation of four lineages between 1999 and 2001 was followed by a slight decrease in the genetic diversity and the emergence of a new and diversified lineage since 2004-2005. In North America, one dominant lineage has circulated since 2006. Rapid genetic diversification and extinction processes seem to occur for H7 IA viruses, concordant with surveillance studies that have related strong temporal differences of H7 subtype prevalence in waterbird populations in both Europe and North America [65,66]. Along with previous studies, we suggest that population immunity could play an important role for these epidemiological fluctuations and be responsible for rapid changes in the relative genetic diversity of H7 IA viruses in wild waterbirds [65,67].

Virus spillover to wild birds has been documented many times in association with the circulation of the HP H5N1 IA viruses in Southeastern Asia [24-26]. Introduction of HP viruses from domestic to peri-domestic and wild bird species often occurs in areas where the circulation of these viruses is endemic in domestic birds, and has been suggested as a mechanism of perpetuation of HP IA during and potentially between outbreaks [68,69]. This hypothesis however lacks epidemiological evidence, especially when considering the potential role of domestic ducks as reservoir for HP IA viruses [70,71]. After spillover to wild waterbirds, HP IA virus spread has been shown to be limited in space and time and dependent on various host and environmental factors, such as distance traveled between migratory stopovers, population density, air temperature or virus persistence in aquatic habitats [72-76].

For H7 IA viruses (both LP and HP), we did not detected domestic-to-wild bird transmission with long-term circulation of the introduced virus in wild waterbird populations. Although this could be related to the relatively low number of sequences available from wild birds, this could also reflect adaptations to domestic hosts that result in reduce fitness after spillover in wild waterbird populations [77]. Such hypothesis has been proposed to explain the absence of long-term circulation of HP viruses in wild waterbird populations [78] and could be extended to LP IA viruses that had undergone significant adaptations to domestic hosts and environments, precluding persistence in the wild when a spillover occurs.

### Host shifts and population dynamics in domestic birds

Wild-to-domestic bird transmission has been previously documented and includes the detection of LP ancestral virus of the Asian HP H5N1 viruses [23]. In the present study, ten independent host shifts (wild-to-domestic birds) were detected for H7 IA viruses. Local circulation of a LP H7 viruses in wild waterbirds has been shown to occurs prior to the introduction in poultry (e.g. [21,37]). Wallensten *et al*. also suggested that the increase LP H7 virus prevalence in waterbirds could favor introduction in poultry and affect the risk for emergence of HP strains [66]. In this study, we observed a slight increase of the genetic diversity of viruses circulating in wild waterbirds in Europe since 2004-2005, followed by three independent introductions in domestic birds. Interactions between IA virus prevalence in wild waterbirds, changes in the genetic diversity of viral populations and introduction in domestic birds need to be further studied; such information could provide a basis to estimate the risks of emergence of IA virus in poultry. In addition, local surveillance of LP H7 viruses in wild waterbirds has the potential to provide critical information for the development of efficient vaccines protecting poultry against the emergence of highly pathogenic viruses, as proposed by Sakabe *et al*. [79].

Highly pathogenic H7 IA viruses have circulated for extended periods of time in domestic birds in Pakistan and Australia. In Australia, there was evidence of genetic reassortment, in particular with different NA subtypes [32,33]. Because of the very low number of isolates available in this long time period, it is not possible to determine if the H7 HA genetic lineage represents an endemic circulation of virulent viruses or is the result of multiple introductions of LP viruses that evolved into HP forms.

In Pakistan, HP H7 IA viruses have also circulated for an extended period of time, as the result of poor vaccination or eradication strategies before 2002-2003 [35]. The emergence of these viruses have occurred after 1993 with a co-circulation of two or three major genetic sub-lineages. In addition, H9N2 IA virus also have been detected in poultry infected with HP H7N3 virus with evidence of genetic reassortment between the two viruses [35]. Such a pattern of emergence of HP viruses, with the co-circulation of several genetic lineages and reassortments with other circulating LP viruses (in particular H9N2) is similar to the one observed for HP H5N1 in Southeastern Asia [23,49,80,81].

In live bird markets in the USA, rapid changes in the relative genetic diversity have occurred, with peaks of diversity reached every 2-3 years, suggesting diversification and extinction processes for different sub-lineages of H7 HA. These changes could have been driven by eradication procedures that were initiated to control the outbreak. The case of the H5N2 outbreak in poultry in Mexico, with the diversification and co-circulation of multiple genetic sub-lineages after vaccination, has shown that control and eradication procedures can act as a strong selective pressure for IA evolution [82]. In the USA, the H7 IA virus genetic diversity has also been maintained by genetic reassortments with the NA and NS (nonstructural) gene segments of other IA viruses circulating in live bird markets [38,39]. Finally, no case of HP viruses were detected in this genetic lineage, although evidence of mutations at the cleavage sites were described [39], contrasting with the rapid evolution toward an increased virulence that was observed in Italy, Australia, Pakistan and Canada.

### Emergence of domestic bird-adapted IA viruses

Influenza A viruses exhibit high rates of nucleotide substitutions allowing continuous antigenic changes, favoring escape from population immunity [49,50,83]. In this study, we highlighted that differences in this rate can be obtained when viruses isolated from wild and domestic birds are analyzed separately. Overall, we found higher rates for viruses circulating in wild birds than in domestic birds, however, important differences were observed between genetic lineages in domestic birds (from 0.37 x10^-3 ^to 5.59 x10^-3 ^substitution/site/year). Previous works has suggested that after introduction in poultry the rate of nucleotide substitution could increases significantly (e.g. for H5 HA; [84]). For H7 HA, the opposite trend was observed but we speculate that this rate is highly variable in poultry, most likely depending on the selective pressures from the domestic environment. In wild waterbirds, naturally acquired immunity has a strong importance on IA virus evolution, favoring continuous antigenic changes. In domestic bird populations, rates of nucleotide substitutions could be strongly affected by immunity acquired through vaccination. This would explain the differences observed between outbreaks where no vaccination was performed for several years (e.g. Pakistan) as compared to the ones where intensive control strategies were developed (e.g. USA, Italy), although such trend would needs further investigations to be validated.

During the last century, at least 11 unrelated genetic lineages of HP H7 IA virus would have circulated in domestic birds. As for H5 IA viruses, evolution from LP to HP viruses arose at several occasions, independently [85]. The amino acid sequences of the H7 HA cleavage site is highly polymorphic between genetic lineages. For instance, evidence of recombination between the HA and the NP genes has been shown to be at the origin of the nucleotide insertion in the cleavage site for the Chilean outbreak [86]. In addition to these reports from outbreaks in poultry, experimental infections with different HP H7 strains have confirmed that different amino acid sequences in the cleavage site can lead to similar levels of virulence [30,68,87,88]. Amino acid insertion in the H7 HA cleavage site could therefore be selected for but with little evidence for a specific sequence motif, as the resulting increase in virulence is likely to increase viral fitness compared to wild-bird origin LP viruses. The absence of detection of HP viruses in H7 genetic lineages that circulated for extended periods of time (e.g. in the USA between 1994 and 2006) however suggests that high virulence is not likely to be selected for under all type of bird production systems, and may not always represents an optimum for viral fitness.

As reported by previous studies [49,50,83], the low mean d_N_/d_S _ratios and the detection of numerous negatively selected sites supports that the H7 HA is under strong purifying selection. For viruses circulating in domestic birds, we found several sites under positive selection, most of them located in the receptor binding domain. In particular, the amino acid mutation at position 143 (135 in H3 numbering; [89]) led to the generation of an additional N-linked glycosylation site in the HA1 coding region. N-linked glycosylation sites are recognized to be involved in host cell entry, proteolytic processing and protein trafficking [90]. The amino acid mutation at position 143 was positively selected in three unrelated genetic lineages (USA, Pakistan and Italy during the 1999-2001 outbreak). In addition, a previous study also reported the acquisition of this glycosylation site in unconnected farms in Italy and suggested that strong selective pressure in poultry could favor the simultaneous and rapid acquisition of this amino acid mutation in separate genetic lineages [27]. The location of this amino acid mutation, at the right edge of the receptor binding domain of the HA protein [91], suggests an importance for host infection and provides evidence of selection for increased host specificity in domestic birds. In addition, it has been shown that important amino acid mutations in the receptor-binding site can be fully functional [92], supporting that such structural modifications in the HA protein can potentially increase IA viral fitness in domestic birds. Positive selection for amino acid changes in genetically unrelated lineages supports evidence of parallel evolution in the HA gene of IA viruses in domestic birds. This is likely to result from host shifts and broadly to be a response to the new selective pressures composing domestic bird production systems (e.g. host density, host genetic diversity, population immunity, environmental transmission), and underlines the need to consider host ecology as well as the consequences of ecosystem shifts in IA virus evolution.

## Competing interests

The authors declare that they have no competing interests.

## Authors' contributions

CL conceived the study and analyzed the data. CL and DES wrote the paper. All authors read and approved the final manuscript.

## Supplementary Material

Additional file 1 **Figure S1 Host diversity of the 414 H7 IA viruses included in the analysis**. Unidentified hosts included 'avian', 'fowl', 'non-psitaccine' and 'psitaccine' for domestic birds and 'shorebird', 'gull' and 'duck' for wild birds. 'Others' domestic birds included 'African starling', 'common iora', 'conure', 'fairy bluebird', 'macaw', 'parakeet', and 'parrot'. Click here for file

Additional file 2 **Table S1 Geographic origin of the 414 H7 IA viruses analyzed**. Numbers in parenthesis report HP viruses included in each group. Click here for file

Additional file 3 **Figure S2 Subtype combination frequency of H7 IA viruses for wild (green) and domestic (orange) birds**. Click here for file

Additional file 4 **Figure S3 Detailed maximum likelihood consensus phylogram**. Tip color represents the geographic origin of the viruses: dark blue: Europe; light blue: Australia; green: Asia; yellow: Africa; purple: South America; orange: North America. Internal branches were also colored for monophyletic groups. Viral strain names were colored in red for HP viruses and annotated with a W or D for viruses isolated in wild and domestic birds, respectively. Bootstrap values are noted when superior to 90%. Click here for file

Additional file 5 **Figure S4 Maximum clade credibility tree for the HA of H7 IA viruses isolated in Eurasia, Africa and Australia**. Tip and branch colors represent host origin (wild in green, domestic in orange). Viral strain names were colored in red for HP IA viruses. Main genetic lineages, with information related to virus origin, were highlighted in the right part of the tree. Nodes with posterior probability values superior to 0.9 were annotated, as well as 95% highest posterior density for times of the most recent common ancestor (blue bars). Blue letters (A to H) represent potential host shift events. Click here for file

Additional file 6 **Figure S5 Maximum clade credibility tree isolated for the HA of H7 IA viruses North and South America**. Tip and branch colors represent host origin (wild in green, domestic in orange). Viral strain names were colored in red for HP IA viruses. Main genetic lineages, with information related to virus origin, were highlighted in the right part of the tree. Nodes with posterior probability values superior to 0.9 were annotated, as well as 95% highest posterior density for times of the most recent common ancestor (blue bars). Blue letters (I to N) represent potential host shift events. Click here for file

Additional file 7 **Table S2 Host shift events suggesting an introduction of a H7 IA virus from wild to domestic birds**. ^1 ^evidenced from the analysis of the maximum clade credibility trees (Figures 2-3 and S4-S5); ^2 ^most related virus or genetic lineage identified in wild birds; ^3 ^virus or genetic lineage circulating in domestic birds following host shift; ^4 ^time of the most recent common ancestor (TMRCA) between the domestic and wild lineages; ^5 ^estimated time period of circulation in domestic birds, in years (based on the difference between the last virus isolation event and the TMRCA); ^6 ^TMRCA for the 'A/Chicken/Netherlands HP H7N3' clade. Click here for file
