# Supplementary figures and images for: Host shifts and molecular evolution of H7 avian influenza virus hemagglutinin
Source: Virol J. 2011 Jun 28;8:328. doi: 10.1186/1743-422X-8-328 (PMC3141685; doi:10.1186/1743-422X-8-328)

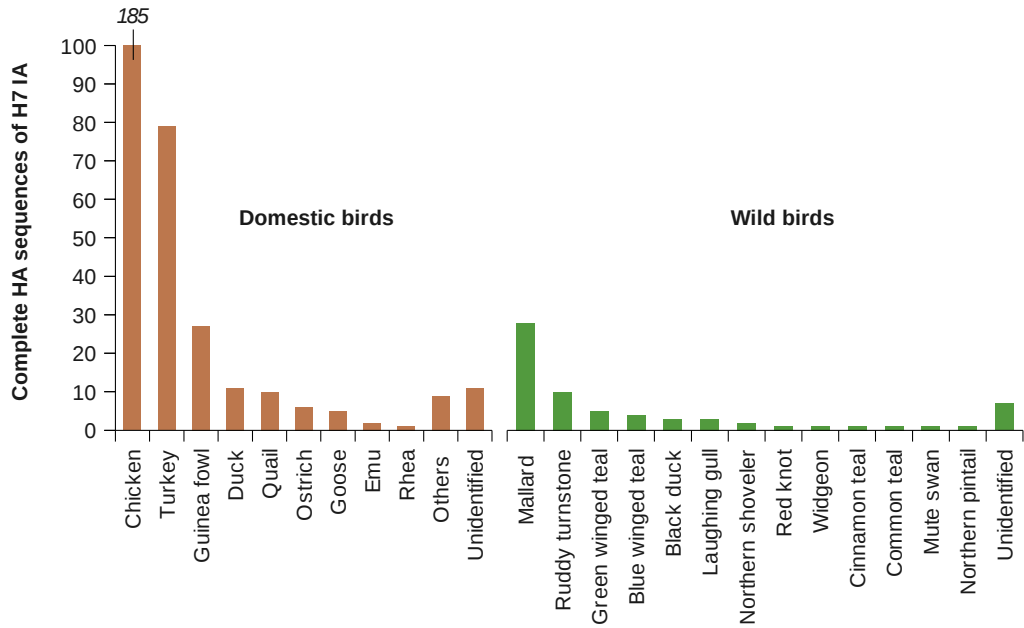

Supplement: Additional file 1 — Figure S1 Host diversity of the 414 H7 IA viruses included in the analysis. Unidentified hosts included 'avian', 'fowl', 'non-psitaccine' and 'psitaccine' for domestic birds and 'shorebird', 'gull' and 'duck' for wild birds. 'Others' domestic birds included 'African starling', 'common iora', 'conure', 'fairy bluebird', 'macaw', 'parakeet', and 'parrot'. [file 1743-422X-8-328-S1.PDF]

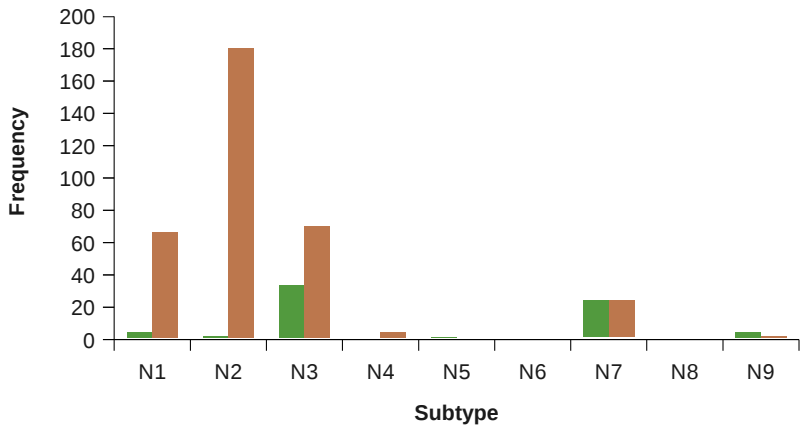

Supplement: Additional file 3 — Figure S2 Subtype combination frequency of H7 IA viruses for wild (green) and domestic (orange) birds. [file 1743-422X-8-328-S3.PDF]

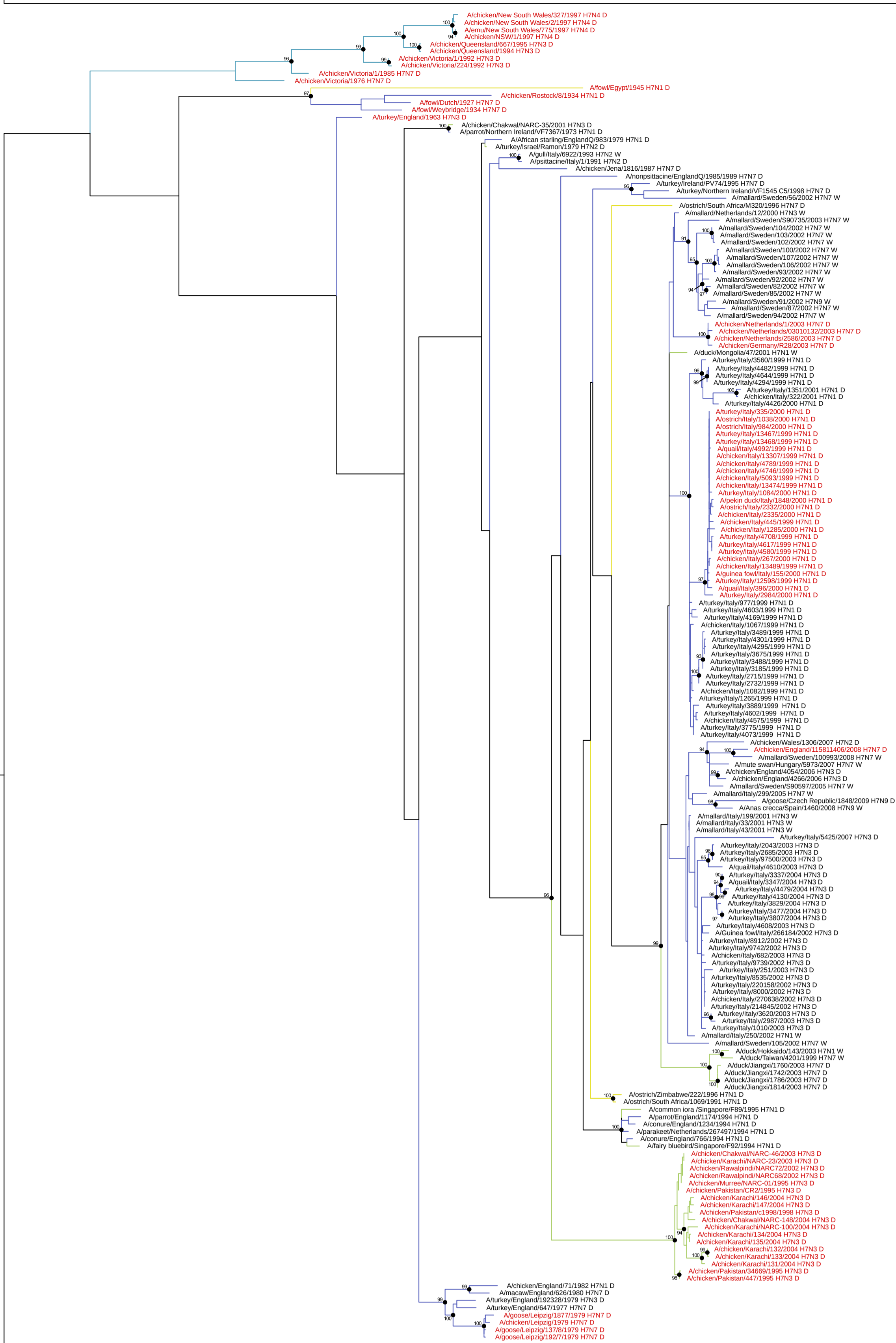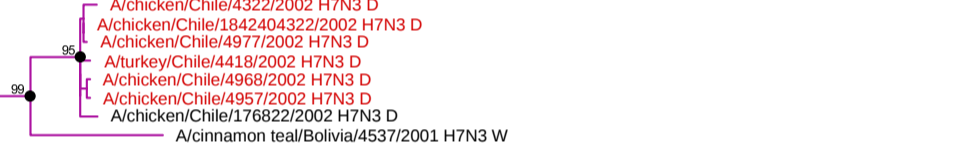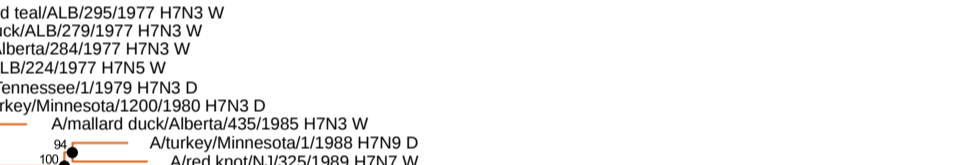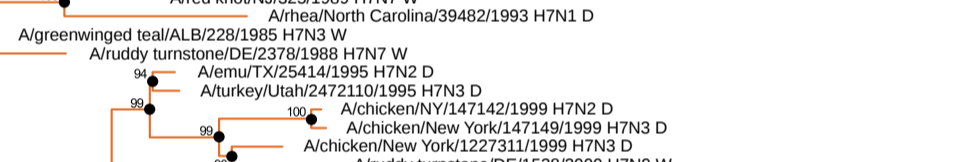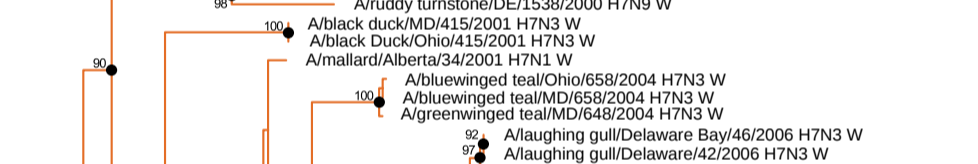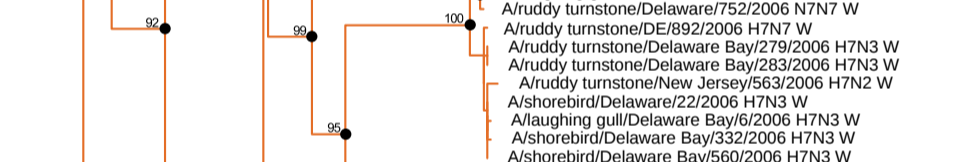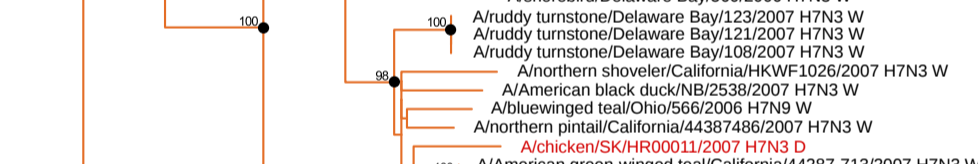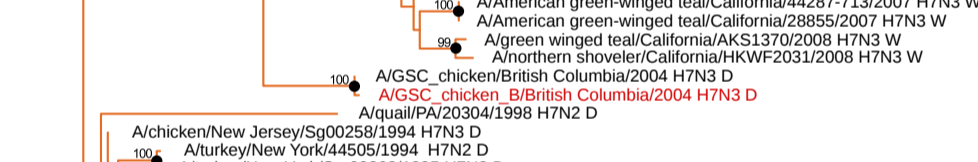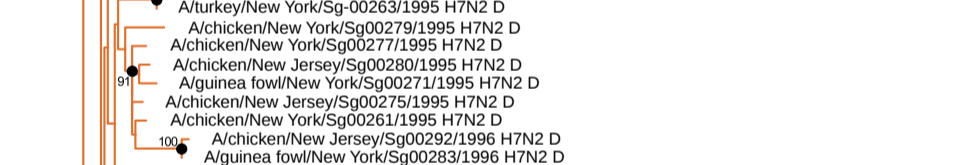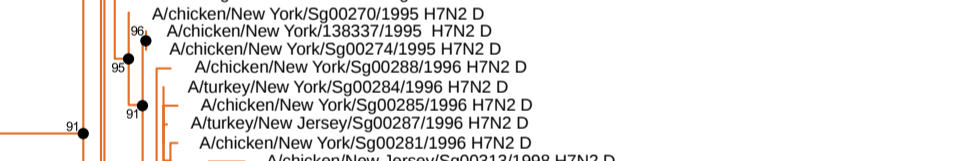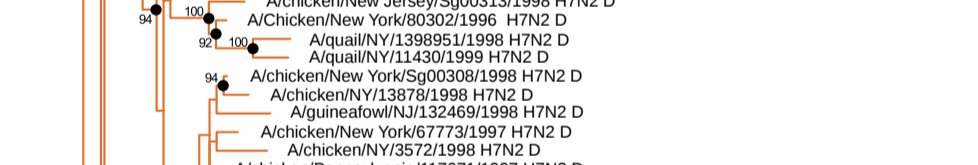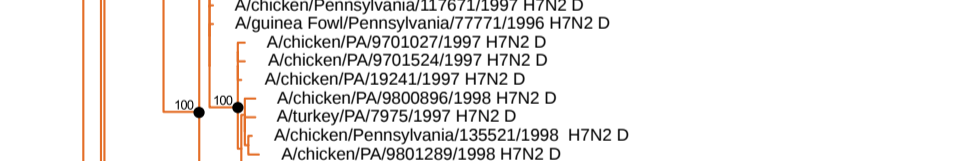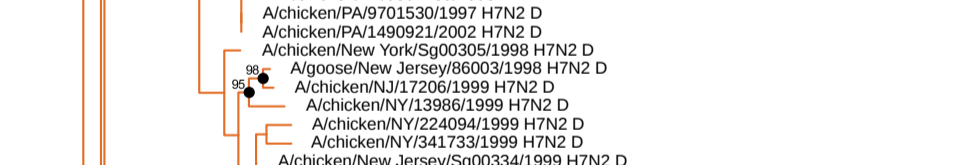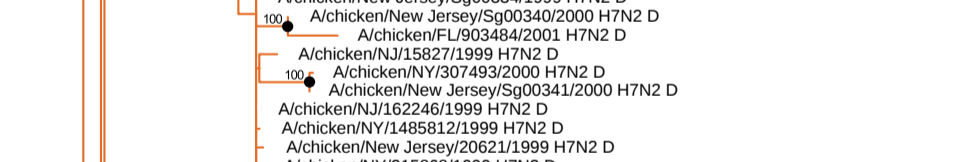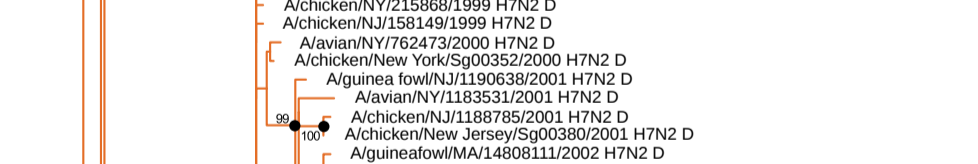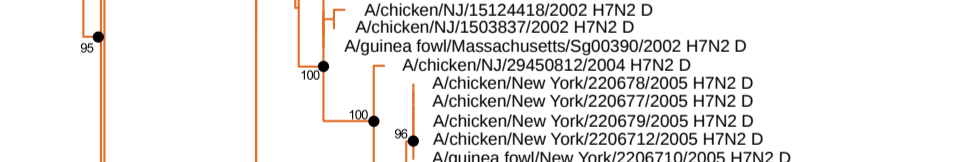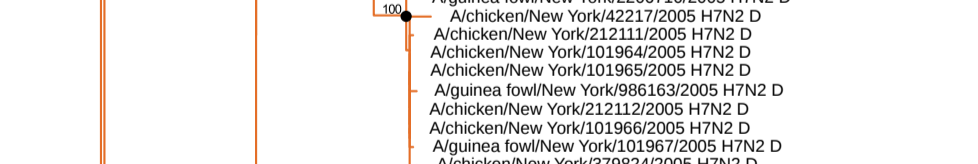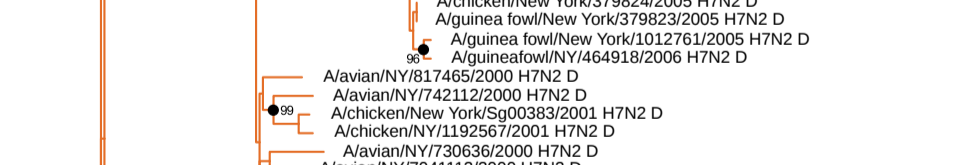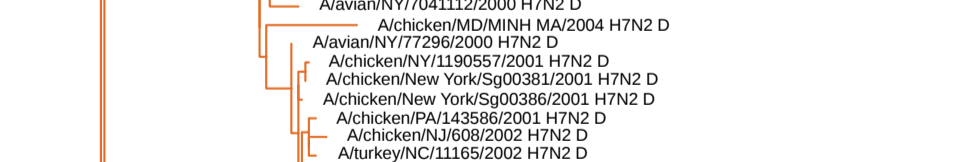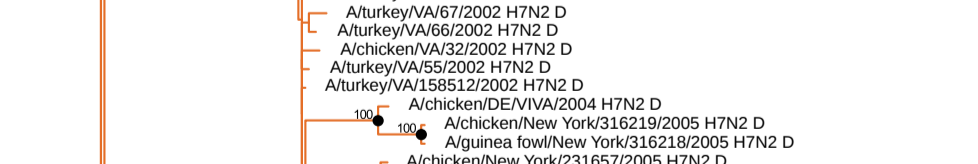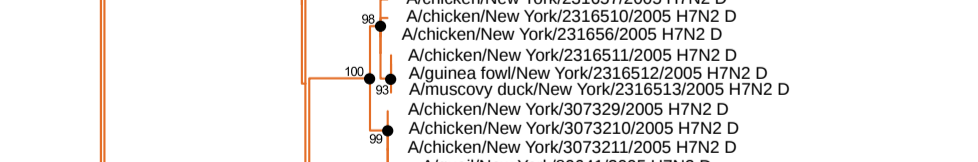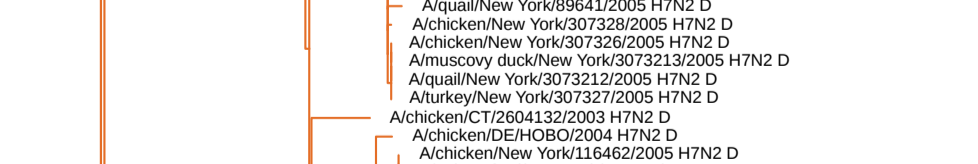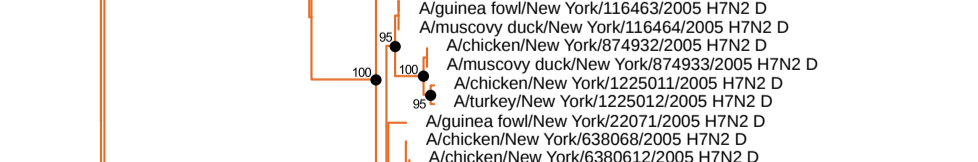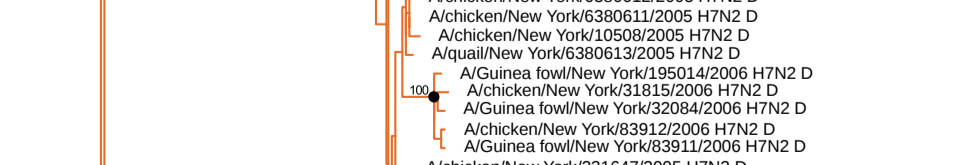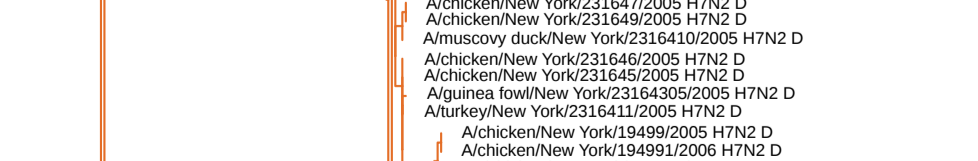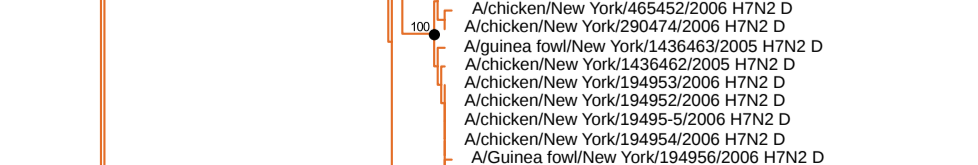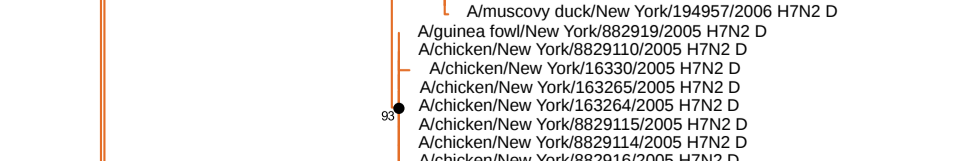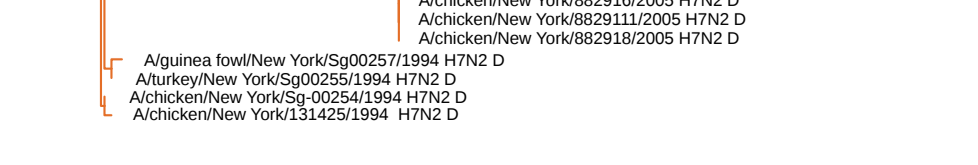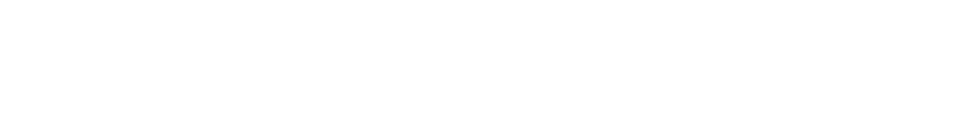

Supplement: Additional file 4 — Figure S3 Detailed maximum likelihood consensus phylogram. Tip color represents the geographic origin of the viruses: dark blue: Europe; light blue: Australia; green: Asia; yellow: Africa; purple: South America; orange: North America. Internal branches were also colored for monophyletic groups. Viral strain names were colored in red for HP viruses and annotated with a W or D for viruses isolated in wild and domestic birds, respectively. Bootstrap values are noted when superior to 90%. [file 1743-422X-8-328-S4.PDF]

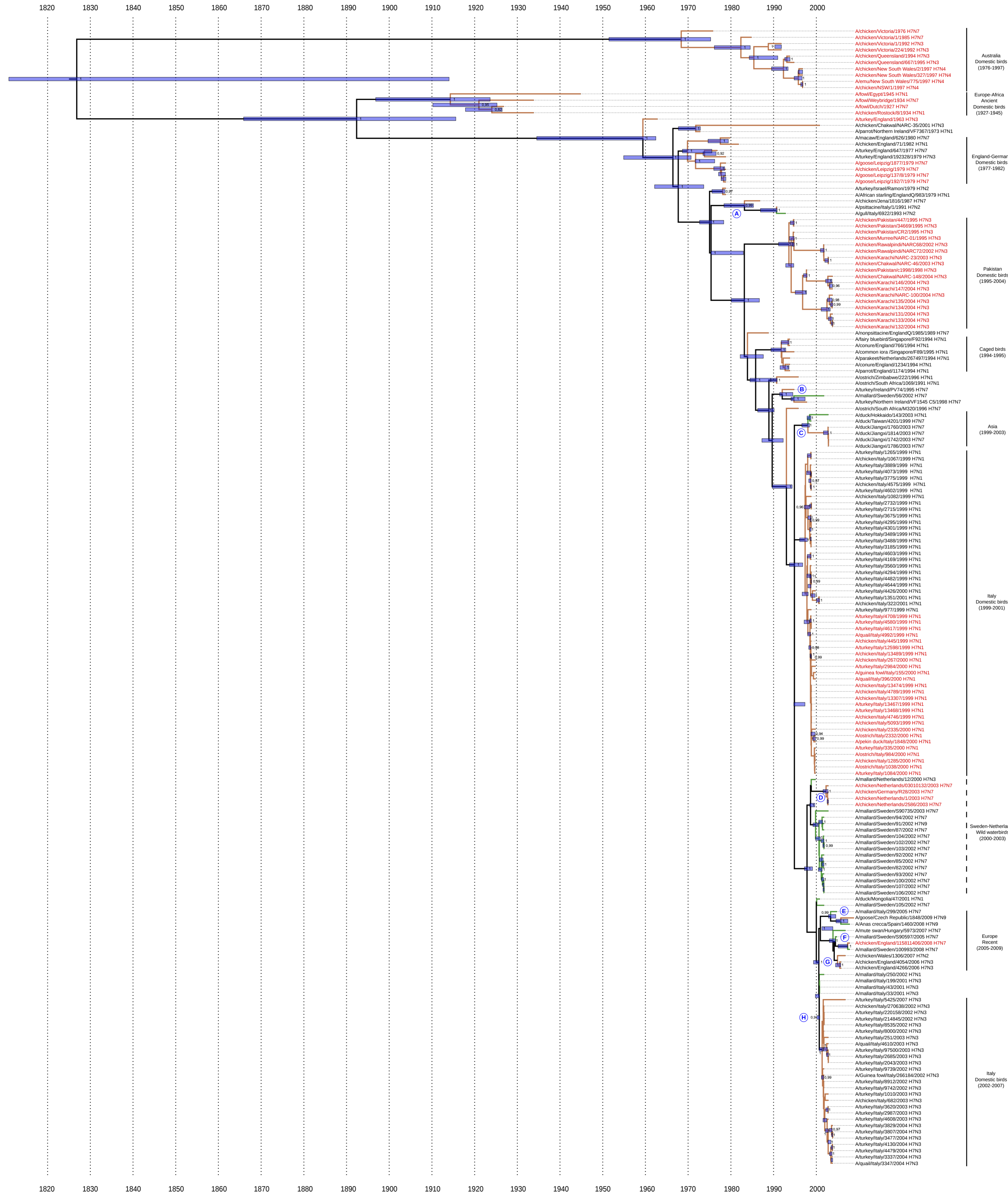

Supplement: Additional file 5 — Figure S4 Maximum clade credibility tree for the HA of H7 IA viruses isolated in Eurasia, Africa and Australia. Tip and branch colors represent host origin (wild in green, domestic in orange). Viral strain names were colored in red for HP IA viruses. Main genetic lineages, with information related to virus origin, were highlighted in the right part of the tree. Nodes with posterior probability values superior to 0.9 were annotated, as well as 95% highest posterior density for times of the most recent common ancestor (blue bars). Blue letters (A to H) represent potential host shift events. [file 1743-422X-8-328-S5.PDF]

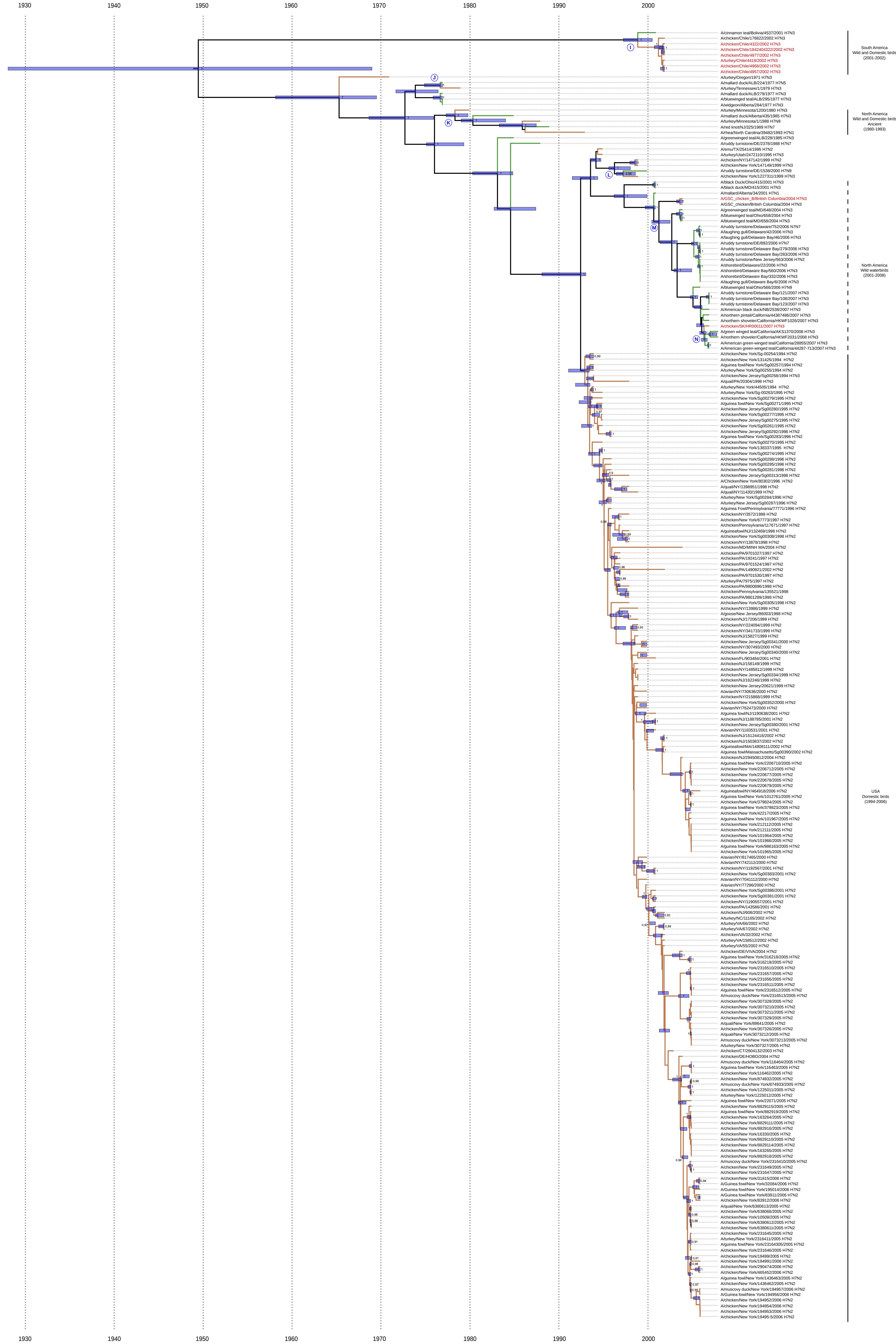

Supplement: Additional file 6 — Figure S5 Maximum clade credibility tree isolated for the HA of H7 IA viruses North and South America. Tip and branch colors represent host origin (wild in green, domestic in orange). Viral strain names were colored in red for HP IA viruses. Main genetic lineages, with information related to virus origin, were highlighted in the right part of the tree. Nodes with posterior probability values superior to 0.9 were annotated, as well as 95% highest posterior density for times of the most recent common ancestor (blue bars). Blue letters (I to N) represent potential host shift events. [file 1743-422X-8-328-S6.PDF]
