# Supplementary material for: Host shifts and molecular evolution of H7 avian influenza virus hemagglutinin
Source: Virol J. 2011 Jun 28;8:328. doi: 10.1186/1743-422X-8-328 (PMC3141685; doi:10.1186/1743-422X-8-328)
Supplement: Additional file 2 — Table S1 Geographic origin of the 414 H7 IA viruses analyzed. Numbers in parenthesis report HP viruses included in each group. [file 1743-422X-8-328-S2.PDF]

	North America	South America	Europe	Africa	Asia	Australia
**Domestic**	188 (2)	7 (6)	111 (39)	4 (1)	26 (18)	10 (10)
**Wild**	37	1	27	0	3	0
