# Supplementary material for: Host shifts and molecular evolution of H7 avian influenza virus hemagglutinin
Source: Virol J. 2011 Jun 28;8:328. doi: 10.1186/1743-422X-8-328 (PMC3141685; doi:10.1186/1743-422X-8-328)
Supplement: Additional file 7 — Table S2 Host shift events suggesting an introduction of a H7 IA virus from wild to domestic birds. 1 evidenced from the analysis of the maximum clade credibility trees (Figures 2-3 and S4-S5); 2 most related virus or genetic lineage identified in wild birds; 3 virus or genetic lineage circulating in domestic birds following host shift; 4 time of the most recent common ancestor (TMRCA) between the domestic and wild lineages; 5 estimated time period of circulation in domestic birds, in years (based on the difference between the last virus isolation event and the TMRCA); 6 TMRCA for the 'A/Chicken/Netherlands HP H7N3' clade. [file 1743-422X-8-328-S7.PDF]

	Wild birds			Domestic birds			Estimated circulation time	
Event[1]	Strain name or genetic lineage[2]	Subtype	Virulence	Strain name or genetic lineage[3]	Subtype	Virulence	TMCRA[4]	Period[5]
C	A/Duck/Taiwan/4201/1999	H7N7	LP	A/Duck/Jiangxi/1760/2003 (China)	H7N7	LP	1998 [1997-1999]	5
D	A/Mallard/Netherlands/12/2000	H7N3	LP	A/Chicken/Netherlands/03010132/2003	H7N7	HP	2002 [2002-2003][6]	1
E	A/Anas crecca/Spain/1460/2008	H7N9	LP	A/Goose/Czech Republic/1848/2009	H7N9	LP	2006 [2005-2008]	3
F	A/Mallard/Sweden/100993/2008	H7N7	LP	A/Chicken/England/115811406/2008	H7N7	HP	2006 [2005-2008]	2
G	A/Mute swan/Hungary/5973/2007	H7N7	LP	A/Chicken/Wales/1306/2007	H7N2	LP	2004 [2003-2005]	3
H	Mallard, Italy, 2001	H7N3	LP	Italy, Domestic birds, 2002-2007	H7N3	LP	2001 [2000-2001]	6
I	A/Cinnamon Teal/Bolivia/4537/2001	H7N3	LP	Chile, domestic birds, 2001-2002	H7N3	LP, HP	1999 [1997-2001]	3
J	A/Mallard duck/ALB/224/1977	H7N5	LP	A/Turkey/Tennessee/1/1979	H7N3	LP	1976 [1975-1977]	3
M	A/Mallard/Alberta/34/2001	H7N1	LP	A/GSC_chicken/British Columbia/2004	H7N3	LP, HP	2001 [2000-2001]	3
N	American Green-winged Teal, California, 2007	H7N3	LP	A/Chicken/SK/HR00011/2007 (Canada)	H7N3	HP	2006 [2006-2007]	1
